# Computational cancer biology: education is a natural key to many locks

**DOI:** 10.1186/s12885-014-1002-2

**Published:** 2015-01-15

**Authors:** Frank Emmert-Streib, Shu-Dong Zhang, Peter Hamilton

**Affiliations:** Computational Biology and Machine Learning Laboratory, Center for Cancer Research and Cell Biology, School of Medicine, Dentistry and Biomedical Sciences, Faculty of Medicine, Health and Life Sciences, Queen’s University Belfast, Belfast, Lisburn Road, Belfast, UK; Center for Cancer Research and Cell Biology, School of Medicine, Dentistry and Biomedical Sciences, Faculty of Medicine, Health and Life Sciences, Queen’s University Belfast, Lisburn Road, Belfast, UK

**Keywords:** Cancer, Computational biology, Genomics data, Computational oncology, Computational genomics, Statistical genomics, Systems medicine

## Abstract

**Background:**

Oncology is a field that profits tremendously from the genomic data generated by high-throughput technologies, including next-generation sequencing. However, in order to exploit, integrate, visualize and interpret such high-dimensional data efficiently, *non-trivial* computational and statistical analysis methods are required that need to be developed in a problem-directed manner.

**Discussion:**

For this reason, computational cancer biology aims to fill this gap. Unfortunately, computational cancer biology is not yet fully recognized as a *coequal* field in oncology, leading to a delay in its maturation and, as an immediate consequence, an *under-exploration* of high-throughput data for translational research.

**Summary:**

Here we argue that this imbalance, favoring ’wet lab-based activities’, will be naturally rectified over time, if the next generation of scientists receives an academic education that provides a fair and competent introduction to computational biology and its manifold capabilities. Furthermore, we discuss a number of local educational provisions that can be implemented on university level to help in facilitating the process of harmonization.

## Background

The origin of molecular oncology is generally traced back to 1975 when Varmus and Bishop discovered that, what is now called *proto-oncogenes*, can initiate cancer in normal cells [1,2]. Deservedly, their finding was awarded with the Nobel price in Physiology or Medicine in 1989. This milestone of cancer research (Nature milestones 15) triggered a myriad of studies leading to an enormous accumulation of knowledge about molecular and cellular mechanisms of many different types of cancer. Here, it is important to note that all of these investigations were based on technologies and wet lab techniques known *prior* to the sequencing of the human genome [3,4].

Interestingly, aside from the main result of the the Human Genome Project, which was to provide the first draft of the human DNA, it helped powerful highthroughput technologies to emerge [5]. These technologies changed the face of biology and medicine rapidly in a profound way. A consequence of these technological innovations is that, nowadays, we are capable of generating high-dimensional transcriptomics, proteomics, metabolomics and imaging data that are containing measurements of thousands and even millions of molecular variables [6-10]. For this reason, in the post-Human Genome Project era, we are blessed with the ability to generate genome-scale data from many molecular and cellular components allowing us to gain insights into the pathogenesis of cancer and causal molecular mechanisms, at least in principle. Some examples for success stories can be found in the following studies [11-16] that demonstrate impressively the power and benefit of computational approaches by providing automatic, consistent and robust methods to extract information from high-dimensional data. Despite the fact that some of these papers have been already published over 10 years ago, they are still widely cited and influence contemporary work. We would like to point out that some of the papers listed above are not specific to cancer research but have a much wider impact, e.g., [11,15]. Unfortunately, the practical transition from ‘data’ to ‘information’ and ‘knowledge’ turned out to be in general a major hurdle and we are currently still struggling to find the ‘right’ approaches and methods for this problem.

An important question in this context is if computational cancer biology is a coequal field in oncology, similar to other sub-areas specialized in particular cancer types, e.g., breast cancer, lung cancer or lymphoma, or if it is merely an ‘auxiliary’ field. Here, we use the term *computational cancer biology* or *computational oncology* to aggregate quantitative fields with oncology to establish computer-driven approaches, but we are well aware that other terms are sometimes used as well. Although, many may be willing to attest computational cancer biology a similar status as other fields, this liberalism comes swiftly to an end if it comes to concrete actions. For example, in terms of funding and the number of employed scientists, computational cancer biology is currently certainly not at eye level with other fields in oncology. Examples for this can be found, e.g., in the MRC strategic plan 2009-2014 that mentions in a 44 page document the term ‘computational’ just once and ‘bioinformatics’ only twice. Similar results can be found for the NCI (The National Cancer Program: Managing the Nation’s Research Portfolio - 2013) of the NIH (‘computational’ × 1, ‘bioinformatics’ × 5) in a 88 page document. Also the number of awarded Training Fellowships by the MRC for Bioinformatics, Biostatistics and Methodology was together only 12 from to a total of 92 awarded Fellowships (13*%*) in all fields in 2011/12 (Annual Report and Accounts 2011/12) requiring only 7*%* of the total Fellowship budget. In contrast, 18 (19*%*) non-clinical Fellowships were awarded consuming 45*%* of the total budget.

This imbalance is also reflected in the number of research groups. For instance, the Cancer Research UK Cambridge Institute, a leading UK institution, employes 20 research groups of which only 2 focus on computational biology. Similarly, at the Center for Cancer Research and Cell Biology at the Queen’s University Belfast (UK) we have 38 research groups in total, but only 3 computational labs. This is also observable in institutions of other countries. For instance, the German Cancer Research Center in Heidelberg has only 9 of its over 90 research groups with such a focus and the Dana-Farber Cancer Institute in Boston (USA) employes 16 faculty member in its Department of Biostatistics and Computational Biology, whereas the Department of Cancer Biology and Department of Medical Oncology have over 200 faculty members.

A similar imbalance can be found in the scientific literature. For instance, in journals like Nature and Science one finds rarely articles that focus on computational cancer biology *without* analyzing original data at the same time, even though profound insights are possible from rigorously reanalyzing existing data, as one can see in [17-19]. Specifically, within the last year Nature/Science published 190/96 contributions about cancer research of which only 5/1 had a strong computational biology component.

## Discussion

### Computational cancer biology as coequal field in oncology

One could wonder why one would like to establish computational cancer biology as a coequal field in oncology at all? The answer to this question is also the cause of the problem we are facing in the post-Human Genome Project era: Data!

Specifically, the possibilities opened nowadays by a number of different high-throughput technologies [20], including next-generation sequencing, to generate ‘big’ genomics data is, without a doubt, an enormous opportunity for medicine and pharmacology in general to elucidate systematically the molecular origin of pathologies, nosology and drug mechanisms [21,22]. On the other hand, due to the novelty of these technologies, but also the general availability of high-dimensional data with a complex correlation structure, methodological developments are limping far behind our data generation abilities. These problems can only be overcome by the development of dedicated analysis approaches because the amounts of data are even expected to further grow [23].

One problem in this context is that oncology as a field does, traditionally, not bring up quantitative scientists, but is mainly concerned with wet lab work and clinical trials. Although, also these subjects require quantitative analysis methods, these are certainly not comparable to the demands triggered by next-generation sequencing (NGS) data, not to mention the integration of different data types. For this reason, oncology needs to be complemented with expertise that comes from *outside* in order to deal appropriately with these novel data types. Here, by *outside* we refer to fields like statistics, computer science, physics or signal processing that deal quantitatively with different aspects of data and that developed a long-standing history of expertise and success [24,25]. This expertise is captured in the field computational cancer biology.

### A naturally induced shifting

Potentially, there are many ways how one could counteract this imbalance. However, a rather natural way is by means of education. Specifically, at the Center for Cancer Research and Cell Biology (CCRCB) at the Queen’s University Belfast, we are organizing since a couple of years a summer research program for students. This program is open to BSc students and senior high school (secondary school) students and runs usually between six and eight weeks throughout the summer months. During this time, the students are distributed to the separate research groups, including computational biology, at the Center according to their interests and aspirations.

A reflection of the value the students attribute to computational cancer biology can be seen from a recent poster competition organized by the CCRCB and Cancer Research UK (CRUK). The task the students were given was to prepare a poster conveying the following information:

Imagine you are the Marketing Manager for the Centre for Cancer Research and Cell Biology (CCRCB) at Queen’s University Belfast. Your role is to promote the work of the CCRCB and Belfast Cancer Research UK Centre throughout Northern Ireland.

**Figure 1:**
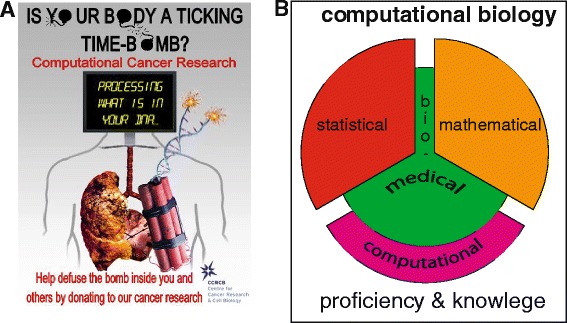
**The winning poster contribution by Aaron Carlisle for a competition organized by the CCRCB & CRUK (A) and the constituting parts of computational biology (B).**

As a result from this competition the winner Aaron Carlisle, a student from Dromore High School (UK), prepared the successful poster, shown in Figure 1A, thematizing the need for computational approaches in cancer research. Among all the topics that could have been selected, e.g., breast cancer, lung cancer or leukaemia, Aaron chose to promote the CCRCB based on research conducted in computational cancer research, because he realized that, no matter what specific type of cancer we are dealing with, it requires *always* a computational approach. As such:

Computational cancer biology is the common denominator of modern oncology.

The outcome of this poster competition is very encouraging, because it reflects an intuitive appreciation students have for computational research in oncology. Here by *intuitive* we mean that it is not necessary to understand computational approaches in the very detail in order to realize their importance and necessity. Biologically, this may be seen as adaptability of the student population to environmental pressure in order to improve their fitness. Certainly, the analogy to a self-organizing system has its limitations, however, it may well be that there are no strong political interventions necessary that dictate, e.g., quota for grants, scientific personnel or publications in flagship journals like Nature and Science, prescribing the inclusion of computational cancer research.

### So, what is needed to enhance computational cancer biology?

We think that, in general, there is no doubt about the importance of computational approaches for cancer research and for this reason it will only be a matter of time when computational cancer biology steps out of the shadow of its big brothers and into the light of a broad recognition. However, the establishment of certain boundary conditions will help to enhance this development. Because it is outside of our area of influence to directly change strategic programs of funding bodies, we discuss here only *local* solutions that can be implemented on the School-, Institute- and Department-level.

Education is a key-element in shaping the future of any field. For this reason we consider the exposure of students to ‘computational biology’ on all relevant education levels as a necessary condition to establish a sustainable program in computational cancer biology. Specific elements are:

- Summer internships (summer research program or summer school for BSc, MSc and PhD students)
- Undergraduate training (BSc course)
- Postgraduate training (MSc course or PhD program)
- Postdoctoral training (seminars)
- Mentoring of colleagues (seminars, conferences, workshops)

There are various ways of engagement on these levels, e.g., via a formal establishment of BSc or MSc courses or, less formal, by providing individual modules, lecture series or lectures as a complement to existing programs. It will depend on the local needs of an institution and on the amount of commitment one is willing to make. However, even more important than the quantity of exposure of students to this subject, is the quality with which it is taught. As an example, we just want to briefly remind the reader to the meaning of a p-value and its relation to the sampling distribution of a null hypothesis. Despite the classic nature of this topic, going back to Fisher and Neyman, it causes unease in generations of students, because of two reasons. First, the topic itself is not trivial at all. Second, hypothesis testing finds such a wide-spread application, in essentially every scientific field that generates data, that the number of qualified scientists who can confidently teach such subjects is outnumbered by an order of magnitude by application focused researches. For computational approaches this means that before one can sit in front of a computer to use it for a problem one needs to understand what one wants to do, which does not require a computer. This is intimately connected to the usage of software packages that are capable of solving particular data analysis problems.

On each of the above mentioned education levels, it is necessary to teach the students four key skills and knowledge, and their mutual interplay:

- Computational proficiency
- Statistical proficiency
- Mathematical proficiency
- Biomedical knowledge

It is part of the problem that ‘computational biology’ is not one-dimensional, but interdisciplinary composed of different skill sets that are traditionally rooted in different subjects (computer science, statistics, physics, signal processing, applied mathematics, biology and medicine). However, this interdisciplinary character of computational biology is a major factor of its strength! Briefly, computational proficiency ensures independency of available software and enables the implementation of any desired algorithm for analysis or visualization. Statistical and mathematical proficiency allow to design or adapt methods in a problem-directed manner so they interrogate data in a way to extract important and sensible biological information. Finally, biological knowledge is the glue that connects the other parts together; see Figure 1B for an overview. Hence, education is a natural key that can unlock many doors and only if we open all (four) of them, computational cancer biology emerges.

### Practical implementations

The above outline gives only the scaffold of training programs and we would like to emphasize a number of key features that appear for us imperative in order to improve upon many existing programs. First of all, there is a crucial difference between proficiency and knowledge. For knowledge the emphasize is more on facts and information about a problem, whereas proficiency is centered around skill sets and their application. For instance, computational proficiency cannot be acquired by memorizing only the syntax of commands of a programming language. Instead, a programming language needs to be practiced hands-on in the context of sensible problems. Furthermore, a programming language needs to be taught that can be actually applied for analyzing real biological data and not just for showing toy examples. We think that the statistical programming language R [26] is a good choice because, currently, it can be considered as the gold standard in computational biology and biostatistics and is widely used.

Second, for acquiring statistical and mathematical proficiency it is necessary to connect statistical and mathematical problems with their computational implementation. For instance, the teaching of non-parametric hypothesis tests will lead to a deeper understanding of statistical inference in general and specifically the interplay between a test statistic and a sampling distribution. Such a level of understanding cannot be achieved by using ‘point and click’ software packages because they are hiding all practical detail levels resulting in an abstract black-box function. As such, the practical implementation in a programming language of a statistical model can be seen as de-black-boxing of the model.

Third, all statistical models are based on a basic understanding of analysis, linear algebra and probability theory. Hence, without proficiency in these mathematical fields no statistical proficiency can be achieved. This implies that shortcuts in teaching statistical models by avoiding a deeper discussion about the former fields will corrupt a statistical understanding and its proficiency.

Fourth, in order to implement the above measures one needs to schedule sufficient amount of time in the educational and training curriculum. That means, based on the background of students one needs to assess the realistic need for their training to bring them up to the desired proficiency and knowledge level in computational biology. Frequently, the scheduled time framework is over optimistically chosen resulting in frustrated students and instructors alike. Computational biology is a difficult subject and if the training of students does not lead to the desired outcome the objectives of the training are not met and the capabilities of conducting state-of-the art data analyses is severely impaired.

### Accepting limitations

There is one additional point we consider worth emphasizing individually. For this reason, let’s consider the following situation: You are hungry but you cannot cook. What do you do? There are three common options. First, you go out to a restaurant. Second, you learn how to cook. Third, you buy a microwave ready meal. It is immediately clear to everyone that by pursuing the third option you cannot open a restaurant. Also, the quality of your learned cooking skills may not be far above a ready meal if you do not put in the necessary effort.

In the context of computational biology, this example translates as follows: In the first case, there is a facility that solves the data analysis problem for you. In the second case, you receive training, as discussed in the previous sections. The third case corresponds to the acquiring of propriety software that provides you with ready solutions for standard problems.

For biomedical research including oncology that involve patients, it seem imperative that only ‘chefs’ should analyze data and the transition from someone who cannot cook to a chef takes many years. That means, the acquired expertise of trainees and students needs to be fairly assessed and limitations need to be acknowledged in order to avoid later severe problems, e.g., with respect to the reproducibility of the results.

### Responsibility of funding agencies

We do not want to finish this article without remarking that also research councils, e.g., the Medical Research Council (MRC, UK), the National Institutes of Health (NIH, USA) or the European Research Council (ERC, Europe), bear a responsibility to actively influence the evolution of computational biology that should complement the above educational initiative. For instance, the computational biology expertise on grants that propose a significant generation of data should be an enforced requirement to ensure a sound experimental design and analysis of the data. Furthermore, the funding of PhD studentships and Postdoctoral Research Associates could be stirred toward the computational data analysis rather than data generation because the availability of large public data repositories allows to work on biomedical problems without the need to generate data [27].

It is clear that immediate answers to many urging questions cannot be given instantly. For this reason it is commendable that the FT7 program supports the European Network CASYM - COORDINATING ACTION SYSTEMS MEDICINE (https://www.casym.eu/), as an initiative to *develop strategies* for a realization of systems medicine rather than to provide right away an implementation of commonly agreed measures.

## Summary

In this paper, we advocated the opinion that an efficient and natural means for establishing computational biology in oncology is provided via the implementation of broad educational programs. This could lead within one education-cycle, from BSc via PhD to PDRA - namely within 8-10 years, to an assertion of the field, without the need to reformulate other parts of oncology. Here, it is important to emphasize that education, the key of this process, should take place on *all* possible levels, from BSc students to faculty members, in order to harmonize existing differences between computational biology and other parts of oncology naturally, and the more gaps are between these different levels, the more unstable is the outcome of the educational process.

Finally, we would like to note that the problem discussed in this paper has already found appreciation and CASyM and the Wellcome Trust PhD program in Mathematical Genomics and Medicine are good examples. However, we are far away from a general and broad recognition of computational cancer biology as a coequal field in oncology.

We hope that our contribution, which is meant to initiate a wider discussion of this important issue across all relevant communities involved in cancer research rather than as a remedy, will help to establish efficient measures to make computational cancer biology a powerful force in fighting cancer.
